# The In Vitro Effect of Laser Irradiation (Er:YAG and CO_2_) and Chemical Reagents (Hydrogen Peroxide, Sodium Hypochlorite, Chlorhexidine, or Sodium Fluoride) Alone or in Combination on Reducing Root Caries Bacteria

**DOI:** 10.3390/ijms232415732

**Published:** 2022-12-12

**Authors:** Nitya Reddy, Janina Golob Deeb, Todd Kitten, Caroline K. Carrico, Kinga Grzech-Leśniak

**Affiliations:** 1 Department of Periodontics, School of Dentistry, Virginia Commonwealth University, Richmond, VA 23298-0566, USA; 2 Department of Oral and Craniofacial Molecular Biology, Philips Institute for Oral Health Research, School of Dentistry, Virginia Commonwealth University, Richmond, VA 23298-0566, USA; 3 Department of Dental Public Health and Policy, School of Dentistry, Virginia Commonwealth University, Richmond, VA 23298-0566, USA; 4 Laser Laboratory, Department of Oral Surgery, Wroclaw Medical University, 50-425 Wroclaw, Poland

**Keywords:** CO_2_ laser, *Enterococcus faecalis*, erbium laser, oral pathogens, *Streptococcus mutans*, *Streptococcus sanguinis*

## Abstract

(1) Lasers have been used for the treatment of dentinal hypersensitivity and bacterial reductions in periodontology. The purpose of this in vitro study was to evaluate the effect of Carbon Dioxide (CO_2_) and Erbium-doped Yttrium Aluminum Garnet (Er:YAG) lasers with chlorhexidine (CHX), hydrogen peroxide (H_2_O_2_), sodium hypochlorite (NaOCl), or sodium fluoride (NaF) on the viability of oral bacteria associated with root caries. (2) *Streptococcus mutans*, *Streptococcus sanguinis*, and *Enterococcus faecalis* were grown in Brain Heart Infusion (BHI) broth, diluted to an OD660 of 0.5, and treated with antiseptics with or without simultaneous irradiation with the Er:YAG and CO_2_ lasers for 30 s repeated three times. The treatment groups consisted of 1: no treatment, 2: 0.5% H_2_O_2_ alone, 3: 0.5% NaOCl alone, 4: 0.12% CHX alone, 5: 2% NaF alone, 6: laser alone, 7: laser with 0.5% H_2_O_2_, 8: laser with 0.5% NaOCl, 9: laser with 0.12% CHX, and 10: laser with 2% NaF for both lasers. The microbial viability was determined through plating and viable colonies were counted, converted into CFU/mL, and transformed into log form. The statistical analysis was performed using a two-tailed paired *t*-test. (3) The use of CO_2_ and Er:YAG lasers alone failed to show statistically significant antibacterial activity against any of the bacteria. The only effective monotreatment was CHX for *S. mutans*. The combined treatment of 0.5% NaOCl with Er:YAG produced the greatest reduction in overall viability. (4) The combination of the Er:YAG laser with 0.5% NaOCl resulted in the largest reduction in bacterial survival when compared to monotherapies with antimicrobial solutions or lasers.

## 1. Introduction

Infection of the oral cavity is an important global and public health problem. In particular, root caries has become a significant concern with the increasing lifespan of humans and the longevity of dentition with a reported pooled prevalence of 41.5% [1]. Gingival recession can occur in patients with periodontal disease, resulting in local attachment loss and hypersensitivity of dentin [2]. It can also occur in patients with aggressive tooth brushing habits, thin gingival phenotype, and thin bone of alveolar housing and has been associated with aberrant frenal attachment, mucogingival deficiencies, orthodontic therapy, and positional characteristics of teeth, as well as aging [3,4]. These exposed root surfaces create new environments for microbial colonization and are more susceptible to caries due to better bacterial adhesion to the dentin surface and hypersensitivity of the exposed root surface leading to reduced toothbrushing. Demineralization is twice as rapid on the root surface as it is on the enamel because of its lower mineral and higher organic content compared to enamel [5]. Restorative root caries treatment with adhesive materials can be very challenging due to compromised isolation and access, adhesive properties of the root surface, and the lack of retention in preparations due to root form and anatomy [4].

The main etiology for the onset and progression of root caries is the presence of bacteria and fermentable carbohydrates on the root surface [6]. While the demineralization process for enamel starts at the pH of 5.5, root caries initiation and progression start considerably faster at a pH of 6.4 due to the lower degree of mineralization of cementum and dentin covering root surfaces [7]. Untreated caries causes increasing pain complicated by inflammations of gingiva and pulp and can lead to tooth extraction affecting ability to chew and have a direct impact on oral health, which is related to decreased quality of life [8]. Early bacterial colonizers of the root surface implicated in the etiology of root caries include Gram-positive non-mutans streptococci species such as *Streptococcus sanguinis*, *Actinomyces* spp., *Streptococcus mutans*, *Enterococcus faecalis*, *Rothia*, *Veillonella*, anaerobic Gram-negative bacteria, and the yeast, *Candida albicans* [9]. The microflora associated with root surfaces and carious lesions have considerable bacterial diversity and complexity. The presence of *Enterococcus faecalis*, *Streptococcus mutans,* and *Pseudoramibacter alactolyticus* were found in levels higher than 40% in elderly subjects with root caries. As these bacteria were commonly present in dentinal samples from root caries while rarely present or absent in other categories, *Enterococcus faecalis* was implicated as a bacterium associated with root caries [9].

Laser light affects microbial cell integrity including Gram-positive and Gram-negative bacteria, *C. albicans*, and other periopathogens [10]. The erbium lasers, including the Erbium-doped Yttrium Aluminum Garnet (Er:YAG) laser, coincide with the most optimal absorption peak of water and has reportedly had good bactericidal effects at low energy outputs and have thus been used as an effective alternative method to traditional periodontal scaling and root planning (SRP) [11]. It has been shown to remove the smear layer on the root surfaces without any apparent heat damage [10]. The use of erbium lasers as an adjunct to SRP is favorable [12,13]. When compared to mechanical scaling alone, laser treatment decreases endotoxins and lipopolysaccharides on the root surfaces and increases the growth and adherence of gingival fibroblasts, which may aid in the reattachment of gingival tissues to the root surface [11]. Erbium lasers can also be used for other applications such as deepithelialization, debonding ceramic restorations, and soft and hard tissue surgery [14,15].

The Carbon Dioxide (CO_2_) laser with 10,600 nm is commonly used in dentistry and widely considered the best surgical laser for coagulation during and after surgery [16]. The chromophore for the CO_2_ laser is water, which is similar to the Er:YAG, but differs in that the CO_2_ laser targets water inside soft tissues and does not rely on an external water source as does the Er:YAG. The absorbed CO_2_ laser energy causes the water within the tissue to vaporize and removes tissue through ablation [17].

Oral rinses are used in dentistry for their antimicrobial properties. Chlorhexidine (CHX) has bacteriostatic effects and is used pre-operatively and post-operatively due to its effective reduction in bacterial load [18,19]. Hydrogen peroxide (H_2_O_2_) rinses have been used for many years to help control plaque and oral infections and provide high antiplaque efficacy, decreased gingival inflammation, and oral bacteria counts with no side effects at 1.5% concentration [20]. At 3% concentration, available over the counter, with a maximum contact time of seven minutes, no mucosal irritation was noted in an animal model. The hydroxyl radicals generated by H_2_O_2_ photolysis provide a powerful oxidizing agent capable of inducing oxidative damage to oral bacteria [21].

There has been limited research examining the effect of chemical irrigants in conjunction with lasers on the bacterial counts of species implicated in root caries. The purpose of this in vitro study is to evaluate the effectiveness of using chemical irrigants, H_2_O_2_, NaOCl, CHX, and NaF, in conjunction with the Er:YAG and CO_2_ laser on the bacterial viability of *Streptococcus mutans*, *Streptococcus sanguinis*, and *Enterococcus faecalis* in an effort to identify if they could be used as adjunctive therapies in the treatment of root caries. We hypothesize that the combination of an irrigant with a laser will yield a greater bactericidal effect than either the irrigant or laser alone.

## 2. Results

Under the herein applied parameters, the use of CO_2_ and Er:YAG lasers alone had limited antibacterial effects, but there were significant differences in bacterial survival based on the use of irrigant, laser, and bacteria for all combinations. A summary of the models is presented in Table 1. Table 2 presents the pairwise comparisons for the use of irrigants with and without the laser (laser + irrigant vs. irrigant alone). Table 3 presents the pairwise comparisons for the use of each laser with and without the irrigant (i.e., laser + irrigant vs. laser alone).

### 2.1. CO_2_ Laser

The CO_2_ laser demonstrated a synergistic effect with NaOCl by killing significantly more *E. faecalis* than NaOCl alone (−5.7 logs, adj *p*-value < 0.0001) and significantly more than the CO_2_ laser alone (−7.6, adj *p*-value = <0.0001). None of the other laser and irrigant combinations were effective at reducing *E. faecalis*.

The CO_2_ laser also demonstrated a synergistic effect with NaOCl by killing significantly more *S. sanguinis* than with NaOCl alone (−6.4, *p*-value < 0.0001) or the CO_2_ laser alone (−8.1, *p*-value < 0.0001). None of the other laser and irrigant combinations were effective at reducing *S. sanguinis*.

**Figure 1:**
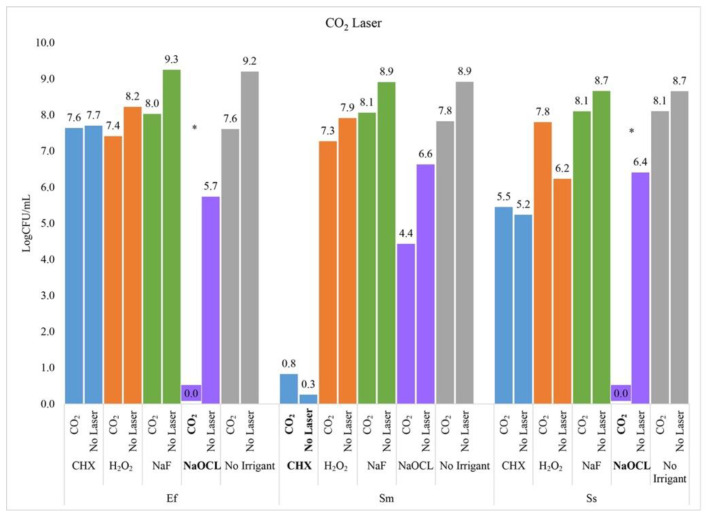
CO_2_ Laser. Asterisk indicates a statistically significant difference between the two groups (i.e., Laser + Irrigant vs. Irrigant Alone) based on Tukey’s adjusted pairwise comparisons.

For *S. mutans*, the only effective treatment was chlorhexidine and there was no additional benefit with the CO_2_ laser (adjusted *p*-value = 1.00) (Figure 1).

### 2.2. Er:YAG Laser

The Er:YAG laser demonstrated a synergistic effect with NaOCl by killing significantly more *E. faecalis* than with NaOCl alone (−6.6 logCFU, adj *p*-value < 0.0001) and significantly more than with the Er:YAG laser alone (−8.2 logCFU, adj. *p*-value < 0.0001).

There was also a synergistic effect between the Er:YAG laser and NaOCl for *S. mutans*, with the combination killing significantly more *S. mutans* than with NaOCl alone (−6.6 logCFU, adj *p*-value < 0.0001) or the laser alone (−8.6 logCFU, adj *p*-value < 0.0001). The chlorhexidine reduced *S. mutans* to undetectable levels independent of the Er:YAG laser (−0.3 logCFU, adj *p*-value = 1).

**Figure 2:**
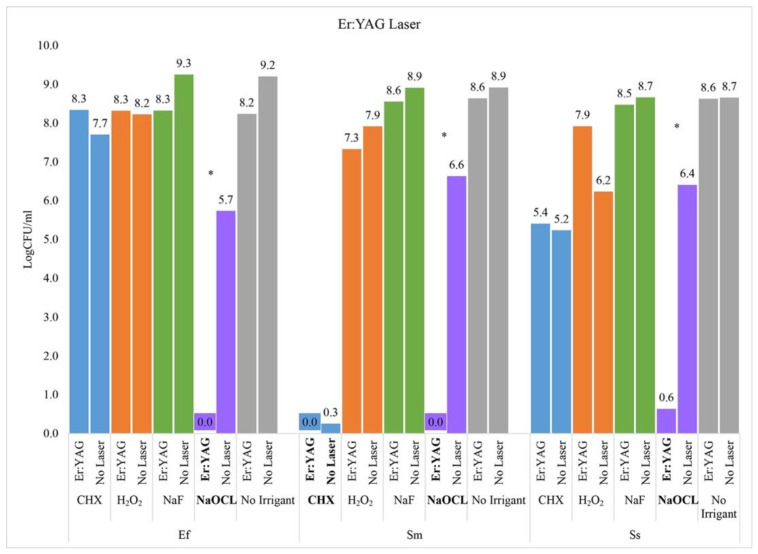
Er:YAG Laser. Asterisk indicates a statistically significant difference between the two groups (i.e., Laser + Irrigant vs. Irrigant Alone) based on Tukey’s adjusted pairwise comparisons.

For *S. sanguinis*, there was a synergistic effect between the Er:YAG laser and NaOCl with significantly less *S. sanguinis* growth combined than with the laser alone (−8.0 logCFU, adj *p*-value < 0.0001) or with NaOCl alone (−5.8 logCFU; adj *p*-value < 0.0001) (Figure 2).

### 2.3. Chemical Reagents

Fluoride did not have any effect alone or in combination with any laser on bacterial viability for *S. sanguinis*, *S. mutans*, or *E. faecalis*. Hydrogen peroxide was only statistically significant in reducing bacterial growth for *S. mutans*, but still did not eliminate all bacteria (−3.2 logCFU, adj *p*-value = 0.0240). CHX was effective as a monotherapy for *S. mutans* reducing the bacterial count to undetectable levels with and without lasers (adj *p*-values = 1) and was statistically significantly more effective on Ss (−5.2 logCFU, adj *p*-value < 0.0001) and *E. faecalis* (−5.2 logCFU, adj *p*-value < 0.0001) when used in combination with the Er:YAG or CO_2_ laser. Sodium hypochlorite 0.5%, proved to have the most extensive effect. NaOCl in combination with Er:YAG resulted in a synergistic effect for all three bacterial species. NaOCl with the CO_2_ laser was effective for *E. faecalis* (−5.7 logCFU, adj *p*-value < 0.0001), and *Ss* (−6.4 logCFU, adj *p*-value < 0.0001) but not *S. mutans* (−2.2 logCFU, adj *p*-value = 0.5227).

### 2.4. Bacterial Strains

The most effective treatment for *E. faecalis* was NaOCl in combination with CO_2_, or the Er:YAG laser, which resulted in undetectable amounts of *E. faecalis* after treatment. Either treatment, NaOCl or the laser alone, was unable to achieve the same level of bacterial reduction as the combination showing the synergistic effects of an antimicrobial with laser treatment. For the CO_2_ laser, NaOCl (5.7, adj *p*-value < 0.0001) or the laser alone (7.6 logCFU, adj *p*-value < 0.0001) resulted in significantly more growth than the combination. The results were similar for the Er:YAG laser and NaOCl combination as compared to NaOCl (5.7 logCFU, adj *p*-value < 0.0001) or the laser alone (8.2 logCFU, adj *p* value < 0.0001).

The most effective treatment for *S. mutans* was CHX alone or combination Er:YAG with NaOCl. The Er:YAG with NaOCl resulted in significantly less bacterial growth than NaOCl alone (−6.6 logCFU, adj *p*-value < 0.0001) and Er:YAG laser alone (−8.6 logCFU, adj *p*-value < 0.0001). While the Er:YAG and CO_2_ lasers provided a synergistic effect in combination with NaOCl, CHX by itself also produced the same result as compared to CHX with Er:YAG with CHX (0.26 logCFU, adj *p*-value = 1) or CO_2_ with CHX (0.57 logCFU, adj *p*-value = 1).

The most effective treatment for *S. sanguis* was CHX or combination NaOCl with the Er:YAG and combination NaOCl with the CO_2_ laser. All these combinations resulted in undetectable levels of *Ss* after treatment. While the Er:YAG and CO_2_ lasers provided a synergistic effect in combination with NaOCl, CHX by itself also produced the same result as compared to the Er:YAG with CHX (0.17 logCFU, adj *p*-value = 1) or CO_2_ with CHX (0.21 logCFU, adj *p*-value = 1).

## 3. Discussion

This study was able to show the effectiveness of the CO_2_ and Er:YAG lasers in conjunction with antimicrobials in reducing the bacterial viability of *E. faecalis*, *S. mutans*, and *S. sanguis*. A combination of NaOCl with Er:YAG laser has shown in vitro synergistic bactericidal effects in reducing the viability and growth of these three bacterial species to undetectable levels. Both lasers as singular treatments did not reduce the bacterial load for any of the three tested species.

Previous studies have confirmed the bactericidal and synergistic effect of the Er:YAG laser when combined with a low concentration of antimicrobials, such as 0.5% NaOCl, 0.5% H_2_O_2_, or 0.03% CHX, on reducing periodontal pathogens, specifically *P. gingivalis*, *F. nucleatum*, and *S. gordonii* [22,23]. Similar laser settings were used in this investigation that could be employed as a supportive periodontal treatment protocol and root caries prevention strategy. The use of 3% hydrogen peroxide with 980 nm diode laser irradiation has been shown clinically effective in reducing the total bacteria count including periopathogens immediately and sustained for 6 weeks, 12 weeks, and 6 months following treatment in >5 mm and >6 mm periodontal pockets with a reduction in red and orange bacterial complexes [24,25]. The results from this investigation support the concept of a non-invasive supportive treatment protocol that can be implemented during periodontal maintenance in high caries risk patients with attachment loss following periodontal disease or therapy.

The lasers used in this study have different clinical indications. When mechanical periodontal therapy using SRP is compared to a combination of both Er:YAG and Nd:YAG lasers, a significant reduction in red and orange complexes was observed for the laser groups [26]. Using the Er:YAG laser resulted in an 85% reduction in periopathogens, whereas combinations of Er:YAG and Nd:YAG observed a 93% reduction and best improvements in clinical attachment level gain, compared to only 46% reduction in the SRP alone group [26]. The current study builds upon the already established evidence of the benefits of lasers in improving microbiological and clinical outcomes in non-surgical periodontal therapy.

The present study did not find any effect of NaF when used as a monotherapy or in conjunction with lasers on bacterial reduction. Previous studies examining the effect of various concentrations of fluorides on *P. gingivalis* and *S. mutans* cultured on titanium disks also did not find a significant decrease in bacterial growth [27] and even reported a slight increase in bacteria growth when a concentration of 1% gel was used [28]. The application of NaF to the surface of the tooth reduces demineralization of the enamel and promotes remineralization. Fluoride-treated teeth exhibit higher pH values by inhibiting bacterial acid production, which contributes to its antimicrobial effect, rather than having a direct bactericidal effect [29].

This study evaluated three bacteria associated with root caries. *S. mutans* and *S. sanguinis* are frequently found in root caries, whereas *E. faecalis* is more commonly associated with endodontic infections [30]. However, a growing number of studies have observed it in carious lesions, periodontitis, and peri-implantitis [31]. Studies have found *Enterococci* species at much higher rates in carious lesions (55.8%) versus caries-free lesions (7%) and have specifically found *E. faecalis* at a much higher frequency than any other *Enterococci* species [32]. *E. faecalis* appears to be associated with root caries disease processes as it was frequently observed in samples from carious roots, but was rarely present or absent in non-carious dentinal root samples [9].

Periodontally treated patients are more susceptible to caries formation due to root exposure and a high incidence of root caries has been observed at 4, 8, 12, and 14 years of maintenance periodontal therapy with approximately two-thirds of patients developing root caries during the first four years [33,34,35,36]. Besides oral hygiene instructions, nutrition counseling, and educating patients, fluoride can be used as an adjunctive treatment to aid in the remineralization of tooth surfaces despite lacking bactericidal effects.

Root caries prevention is of significant importance to periodontists due to its high prevalence and risk rates in 20% of patients referred for periodontal treatment [37]. The findings of the present study suggest that the use of a Er:YAG laser with low concentration of NaOCl can be an effective treatment protocol during maintenance appointments in high caries risk periodontal patients, particularly since there is established use of laser treatments with the present settings and use of chemical irrigants in dentistry [38].

There are several limitations to the present study. The determination of the bactericidal effects of the laser with chemical irrigants was performed in vitro, therefore, its clinical significance in periodontal therapy remains unclear. Additionally, only three bacterial species were evaluated with a small sample size.

## 4. Materials and Methods

### 4.1. Culture Conditions

Three oral bacterial species implicated in the etiology of root caries were used in this study: *S. mutans*, *S. sanguinis*, and *E. faecalis*. They were individually grown but treated in parallel. Freezer stocks (−80 °C) of each bacterial species were obtained and five microliters of a single-use aliquot were used to inoculate 5 mls of Brain Heart Infusion (BHI) broth (Becton–Dickinson; Franklin Lakes, NJ, USA). The inoculum was incubated overnight in an aerobic environment at 37 °C. The optical densities (OD) of the cultures were measured using a spectrophotometer (Genesys 150, Thermo Fisher Scientific, Waltham, MA, USA) at 660 nm (OD660) and normalized to an OD of 0.5. Ten aliquots of 150 μL of each bacterial strain were then transferred to non-adjacent wells of a 96-well plate to facilitate ten treatment groups per experiment. All the treatments were performed in a sterile biological safety cabinet. The chemicals used were added in the form of concentrated stock solutions (3%H_2_O_2_; 5.25% NaOCl; 2%CHX; 75% NaF) directly to the bacterial cultures in the plate. The stock solutions were diluted with sterile water to the concentrations specified per study group. The irradiations with each laser were performed on the appropriate samples. The chemical treatments and laser irradiation were performed one well at a time so that each bacterial culture was in contact with the chemical agent and/or laser for 60 s. Following laser irradiation, each treated sample was diluted 50-fold into fresh BHI broth. Additional dilutions were performed and spread onto BHI plates using an Eddy Jet 2 spiral plater (Neutec Group Inc.; Farmingdale, NY, USA). The plates were incubated for 24–48 h anaerobically at 37 °C. The viable colonies were counted for each plate, converted into CFU/mL, and transformed into log form for statistical analysis.

### 4.2. Laser Irradiation

The Er:YAG and CO_2_ laser was set to normal periodontal clinical settings. The samples were irradiated by a Er:YAG laser at 2940 nm (LightWalker, Fotona, Slovenia), using a 400 µm Varian fiber tip of cylindrical quartz at parameters: 40 mJ; 40 Hz; 1.6 W for 30 s with the 300 µs short pulse duration in contact mode (SP).

An UltraSpeed Smart CO_2_ laser at 10,600 nm (DEKA, Implant Direct, Thousand Oaks, CA, USA) irradiated using a contra-angle attachment and tapered tip at parameters: 50 Hz for 30 s with 0.3 s pulses in direct contact mode. The irradiation was performed with a disinfected aluminum foil barrier to isolate the treated wells from contamination.

### 4.3. Study Group

Group 1a–c: bacteria alone (*S. mutans*, *S. sanguinis*, or *E. faecalis* alone)

Group 2a–c: bacteria (*S. mutans*, *S. sanguinis*, or *E. faecalis*) + H_2_O_2_ (0.5%)

Group 3a–c: bacteria (*S. mutans*, *S. sanguinis*, or *E. faecalis*) + NaOCl (0.5%)

Group 4a–c: bacteria (*S. mutans*, *S. sanguinis*, or *E. faecalis*) + CHX (0.12%)

Group 5a–c: bacteria (*S. mutans*, *S. sanguinis*, or *E. faecalis*) + NaF (2%)

Group 6a–i: bacteria (*S. mutans*, *S. sanguinis*, or *E. faecalis*) + laser (Er:YAG or CO_2_)

Group 7a–i: bacteria (*S. mutans*, *S. sanguinis*, or *E. faecalis*) + laser (Er:YAG or CO_2_) + H_2_O_2_ (0.5%)

Group 8a–i: bacteria (*S. mutans*, *S. sanguinis*, or *E. faecalis*) + laser (Er:YAG or CO_2_) + NaOCl (0.5%)

Group 9a–i: bacteria (*S. mutans*, *S. sanguinis*, or *E. faecalis*) + laser (Er:YAG or CO_2_) + CHX (0.12%)

Group 10a–i: bacteria (*S. mutans*, *S. sanguinis*, or *E. faecalis*) + laser (Er:YAG or CO_2_) + NaF (2%)

Each experiment was performed three times.

### 4.4. Statistical Analysis

The number of surviving colonies of bacterial species were analyzed using ANOVA models. The models evaluated the effects of the combinations of lasers and irrigants on various bacteria species using interaction terms. The post hoc pairwise comparisons were adjusted using Tukey’s adjustment. The significance level was set at 0.05.

## 5. Conclusions

Under the conditions applied herein, lasers combined with low concentrations of commonly used dental chemical agents can provide additional benefits by reducing the bacteria associated with root caries. The use of 0.5% NaOCl in combination with an Er:YAG laser irradiation resulted in the greatest reduction in bacterial viability when compared to monotherapies with antimicrobial solutions or lasers alone. The laser alone was the most effective means for reducing *S. mutans* colonies. NaF had no effect alone or when combined with any of the lasers on bacterial reduction.

## Figures and Tables

**Table 1:** Model results.

	CO_2_		Er:YAG	
	F	*p*-Value	F	*p*-Value
Bacteria	8.09	0.0005	22.64	<0.0001
Irrigant	96.47	<0.0001	132.87	<0.0001
Laser (Y/N)	41.55	<0.0001	42.1	<0.0001
Bacteria*Irrigant	23.74	<0.0001	22.87	<0.0001
Bacteria*Irrigant*Laser (Y/N)	7.62	<0.0001	11.25	<0.0001

Note: Bacteria*Irrigant and Bacteria*Irrigant*Laser (Y/N) indicate the two and three-way interaction terms in the model.

**Table 2:** Pairwise comparisons for the effect of irrigants with and without a laser.

Laser Comparison	Bacterium	Irrigant	∆	SE	*p*-Value	Adj *p*
CO_2_ vs. No Laser	*E. faecalis*	CHX	−0.06	0.78	0.9341	1
		H_2_O_2_	−0.81	0.78	0.2983	1
		NaF	−1.22	0.78	0.1175	0.9993
		NaOCl	−5.74	0.78	<0.0001	<0.0001
		None	−1.59	0.78	0.0419	0.9655
	*S. mutans*	CHX	0.57	0.78	0.4637	1
		H_2_O_2_	−0.64	0.78	0.4103	1
		NaF	−0.85	0.78	0.276	1
		NaOCl	−2.20	0.78	0.0052	0.5227
		None	−1.09	0.78	0.1616	0.9999
	*S. sanguinis*	CHX	0.21	0.78	0.7832	1
		H_2_O_2_	1.57	0.78	0.0451	0.9713
		NaF	−0.56	0.78	0.4721	1
		NaOCl	−6.41	0.78	<0.0001	<0.0001
		None	−0.55	0.78	0.4783	1
Er:YAG vs. No Laser	*E. faecalis*	CHX	0.63	0.76	0.407	1
		H_2_O_2_	0.09	0.76	0.9033	1
		NaF	−0.93	0.76	0.2249	1
		NaOCl	−5.74	0.76	<0.0001	<0.0001
		None	−0.97	0.76	0.2074	1
	*S. mutans*	CHX	−0.26	0.76	0.7378	1
		H_2_O_2_	−0.58	0.76	0.4449	1
		NaF	−0.36	0.76	0.639	1
		NaOCl	−6.63	0.76	<0.0001	<0.0001
		None	−0.28	0.76	0.7128	1
	*S. sanguinis*	CHX	0.17	0.76	0.8237	1
		H_2_O_2_	1.68	0.76	0.0288	0.9233
		NaF	−0.19	0.76	0.8048	1
		NaOCl	−5.77	0.76	<0.0001	<0.0001
		None	−0.03	0.76	0.9688	1

SE = Standard Error; Adj *p* is Tukey’s adjusted *p*-value.

**Table 3:** Pairwise comparisons for effect of laser with and without an irrigant.

Laser	Bacterium	Irrigant		∆	SE	*p*-Value	Adj *p*
CO_2_	*E. faecalis*	CHX	vs. No Irrigant	0.03	0.95	0.9753	1
		H_2_O_2_		−0.20	0.95	0.8376	1
		NaF		0.42	0.95	0.6595	1
		NaOCl		−7.61	0.95	<0.0001	<0.0001
	*S. mutans*	CHX		−7.00	0.95	<0.0001	<0.0001
		H_2_O_2_		−0.55	0.95	0.5619	1
		NaF		0.24	0.95	0.8045	1
		NaOCl		−3.39	0.95	0.0005	0.1051
	*S. sanguinis*	CHX		−2.65	0.95	0.0059	0.5554
		H_2_O_2_		−0.30	0.95	0.75	1
		NaF		0.00	0.95	0.9972	1
		NaOCl		−8.10	0.95	<0.0001	<0.0001
Er:YAG	*E. faecalis*	CHX	vs. No Irrigant	0.10	0.93	0.9145	1
		H_2_O_2_		0.08	0.93	0.9315	1
		NaF		0.09	0.93	0.9275	1
		NaOCl		−8.24	0.93	<0.0001	<0.0001
	*S. mutans*	CHX		−8.64	0.93	<0.0001	<0.0001
		H_2_O_2_		−1.31	0.93	0.1639	0.9999
		NaF		−0.08	0.93	0.9278	1
		NaOCl		−8.64	0.93	<0.0001	<0.0001
	*S. sanguinis*	CHX		−3.22	0.93	0.0007	0.1465
		H_2_O_2_		−0.71	0.93	0.4481	1
		NaF		−0.15	0.93	0.8689	1
		NaOCl		−7.99	0.93	<0.0001	<0.0001
